# Extracts of *Apium graveolens* (Celery) attenuate hepato-renal injury induced by chronic administration of gentamicin in mice through activation of Nrf2-antioxidant signaling pathways 

**DOI:** 10.22038/ajp.2024.25338

**Published:** 2025

**Authors:** Arnaud Fondjo Kouam, Mayelle Mepa Mokam, Eleonore Ngounou, Ferdinand Elombo Kouoh, Rodrigue Fifen, Kerinyuy Juliene Kongnyuy, Elisabeth Menkem Zeuko’o, Nembu Erastus Nembo, Pascal Dieudonné Chuisseu Djamen, Frédéric Nico Njayou, Paul Fewou Moundipa, Emmanuel Acha Asongalem

**Affiliations:** 1 Medical Research and Applied Biochemistry Laboratory, Department of Biomedical Sciences, Faculty of Health Sciences, University of Buea, PO Box 63, Buea, Cameroon; 2 Pharmacology Laboratory, Department of Biomedical Sciences, Faculty of Health Sciences, University of Buea, PO Box 63, Buea, Cameroon; 3 Laboratory of Pharmacology and Toxicology, Department of Biochemistry, Faculty of Science, University of Yaoundé 1, P.O. Box 812, Yaounde 1, Cameroon; 4 Anatomy Laboratory, Department of Biomedical Sciences, Faculty of Health Sciences, University of Buea, PO Box 63, Buea, Cameroon; 5 Laboratory of Animal Physiology, Department of Animal Biology and Physiology, Faculty of Science, University of Yaoundé 1, PO Box 812, Yaoundé, Cameroon; 6 Higher Institute of Health Sciences, Université des Montagnes, P.O. Box 208, Bangangté, Cameroon

**Keywords:** Apium graveolens, Gentamicin-toxicity, Mice, Liver, Kidney, Protection, Antioxidant

## Abstract

**Objective:**

This study aimed at investigating the protective effect of extracts from *Apium graveolens* against gentamicin-induced hepato-renal toxicity.

**Materials and Methods:**

The aqueous and hydro-ethanolic extracts of *A. graveolens* designated respectively as WAG and HAG were tested for their *in vitro* antioxidant activities. Then, their cytoprotective effects were assessed against gentamicin-induced cytotoxicity in primary mouse hepatocytes. Finally, mice were administered with gentamicin (20 mg/kg) and co-treated with HAG for 14 days, and histopathology, biochemical and molecular parameters related to gentamicin-induced toxicity were evaluated.

**Results:**

HAG exhibited outstanding chemical antioxidant activities and preserved hepatocytes from gentamicin-induced cytotoxicity. HAG relieved liver and kidney histopathological and biochemical changes, and enhanced the mRNA level of Nrf2 and its target gene HO-1 in gentamicin-intoxicated mice.

**Conclusion:**

HAG attenuates hepato-renal injuries induced by 14-days administration of gentamicin in mice through the activation of Nrf2-antioxidant signaling pathways.

## Introduction

The application of gentamicin, a popular antibiotic approved for the treatment of bacterial infections, is limited in clinical setting due to serious adverse effects that include ototoxicity, vestibular toxicity characterized by nausea, vomiting and balance disorders (Wu et al., 2002; King et al., 2017). Also, nephrotoxicity and hepatotoxicity, two forms of toxicity that frequently occur to patients who experience prolonged therapy duration have been reported (Chen et al., 2007; Chaves and Tadi, 2022). Indeed, the prominent role of the liver and kidneys in drugs metabolism and excretion particularly sets out these organs to tissue damage (Hayward et al., 2018). Accordingly, considering the vital function played by the liver and kidney in the body, it is necessary to search for a potential antidote to alleviate hepato-renal injury during a long-term gentamicin-based therapy. 

Although the precise mechanisms by which gentamicin induces hepato-renal toxicity is not fully understood, it is suggested that upon long-term administration, gentamicin selectively accumulates in the liver and kidney cortex, inducing oxidative stress with the consequences of oxidation of biological molecules like proteins and lipids (Costa-Silva et al., 1987; Khan et al., 2011). Currently, there is no approved antidote to attenuate gentamicin toxicity. However, several studies substantiate antioxidant based-approach to counter-act adverse effects of gentamicin in the liver and kidneys (Khan et al., 2011; Jannat et al., 2018). Herbal medicines have gained considerable attention due to their therapeutic benefits against chronic diseases (Kalra, 2003; Nasri et al., 2014). It is the case of *Apium graveolens*, better known as “Celery”, which is a common vegetable belonging to the family of Apiaceae. Celery is used in traditional medicine to combat spasm, stomach ache, cardiovascular diseases, gout, urinary tract infection, rheumatic and liver-related diseases (Garcia-Alvarez et al., 2014; Al-Asmari et al., 2017). The phytochemical investigation of extracts from *A. graveolens* showed a rich source of phenolic compounds and vitamins such as caffeic acid, chlorogenic acid, apigenin, and vitamins C and A, which are responsible for its various biological activities (Al-Asmari et al., 2017; Kooti and Daraei, 2017). Celery has also displayed therapeutic effects in animal models of acetaminophen and carbon tetrachloride-induced acute liver failure (Singh and Handa, 1995; Ahmed et al., 2002; Shivashri et al., 2013) However, the ability of celery to confer protection against hepato-renal oxidative injury resulting from long-term administration of gentamicin remains unknown. Accordingly, in the current study, the effect of daily consumption of celery extract as a potential hepato-renal protectant for aminoglycoside-induced oxidative damage in the liver and kidneys was investigated.

## Materials and Methods

### Animals

Healthy male albino mice, weighing between 25-33 g were used. They were maintained in a plastic cage with access to a standard diet and tap water *ad libitum*, under standard laboratory conditions of temperature (about 25±3°C) and 12 hr day light-dark cycle. All the experiments complied with the ARRIVE Guidelines for preclinical animals studies and were approved by the Institutional Animal Care and Use Committee of the University of Buea-Cameroon (UB-IACUC N°14/2023).

### Collection of plant material and preparation of extracts 

Fresh *A. graveolens* (whole celery plant without roots) were purchased from the central market of Buea, South-West Region-Cameroon and authenticated at the Cameroon National Herbarium where the voucher specimen number 43256 KH was assigned. The aqueous and hydro-ethanolic extracts of *A. graveolens* were prepared as previously described (Kouam et al., 2022). 

### Phytochemical screening of the aqueous (WAG) and hydro-ethanolic (HAG) extracts of A. graveolens

The qualitative analysis of HAG and WAG was by detecting the presence of major classes of secondary metabolites such as phenolic compounds, flavonoids, alkaloids, saponins, triterpenes, and tannins using standard methods (Wadood, 2013). The quantitative analysis of phytochemicals was done through the measurement of flavonoids and total phenolic compounds contents using the aluminum chloride and Folin–Ciocalteu methods, respectively as described elsewhere (Kouam et al., 2020). 

### In vitro antioxidant properties of HAG and WAG

The antioxidant properties of different extracts of celery were evaluated using the following assays: 2,2-Diphenyl-Picryl-Hydrazyl (DPPH) (Djeungoue Petga et al., 2023), hydroxyl (HO°) (Kouam et al., 2022), and nitric oxide (NO°) (Kouam et al., 2020) free radical scavenging assays; ferric (Chuisseu et al., 2020) and phosphomolybdenum (Njayou et al., 2015) reducing antioxidant power assays and* in vitro* inhibition of lipid peroxidation (Djeungoue et al., 2023). For each assay, plant extracts (HAG and WAG) and vitamin C (VIC), were tested in triplicate at concentrations range 0.01-100 µg/ml. The detailed procedure for each assay is described in the supplementary file.

### In vitro assessment of the anti-hepatotoxic effect of celery extracts (HAG and WAG) against gentamicin

#### Primary mice hepatocytes isolation

The *in situ* two-step collagenase liver perfusion method as previously described was used for the isolation of primary mice hepatocytes (Djeungoue et al., 2023). Following isolation, hepatocytes were suspended in Dulbecco’s Modified Eagle’s Medium (DMEM) supplemented with L-glutamine (2 mM), NaHCO_3_ (0.5 g/L), penicillin (100 IU/ml), streptomycin (100 µg/ml), Amphotericin B-deoxycholate (Fungizone) (5 µg/ml) and 10% fetal bovine serum and their viability was assessed through trypan blue test. Only hepatocytes isolation with a viability greater than 85% was used. 

### Treatment of isolated hepatocytes

The procedure was done as previously reported (Njayou et al., 2016; Djeungoue et al., 2023). The isolated hepatocytes (≈ 1×10^6^ viable cells/ml) were seeded into a 12-well tissue culture plate (1 ml/well) and maintained at 37°C in an atmosphere of 5% CO_2_. Then, 6 hr after the adherence of cells, the medium was replaced with a fresh medium, and cells were co-treated at the desired concentration with or without gentamicin, plant extracts as well as standard silymarin and cells were incubated for 12 hr followed by biochemical analysis. 

### Evaluation of the cytotoxic effect of gentamicin and celery extracts

For these experiments, hepatocytes were treated only either with gentamicin (0.1-64 mM) or plant extracts (HAG, WAG, or silymarin) (0.1-1000 µg/ml) (Njayou et al., 2016; Djeungoue Petga et al., 2023). MTT assay was performed to assess the viability of cells while the activity of alanine aminotransferase in the incubation medium was measured to evaluate the integrity of the cell membrane. Afterward, the 50% lethal concentration of (LC_50_) of gentamicin was determined from its concentration-response curve.

### Evaluation of the protective effect of celery extracts against gentamicin-induced toxicity in primary mice hepatocytes

Here, cells were co-treated with gentamicin at the predetermined LC_50_, and HAG, WAG, or silymarin. At the end of the incubation period, lipid peroxidation end products, cellular glutathione content, cell viability and membrane integrity were assessed. 

### Evaluation of cell viability and membrane integrity

The viability of hepatocytes was determined through MTT assay as previously described (Kouam et al., 2017). The membrane integrity of hepatocytes was evaluated through measurement of the activity of alanine aminotransferase leakage into the incubation medium, using commercial kits (Cat N° REF_100-0255_CHRONOLAB)

### Assessment of cellular glutathione (GSH) content and lipid peroxidation end-product (malondialdehyde, MDA)

GSH content and lipid peroxidation were quantified as described elsewhere (Djeungoue Petga et al., 2023). GSH and MDA contents were determined using their respective molar extinction coefficient (**ε**_GSH_ = 13,600 M^-1^.Cm^-1^ ; **ε**_MDA_ =1.56 × 10^5^ M^-1 ^.Cm^-1^).

### In vivo assessment of the protective effect of celery extract against hepato-renal injury induced by chronic administration of gentamicin

#### Experimental design

Thirty mice were randomly divided into 6 groups of 5 animals each, and treated daily for 14 consecutive days as follows:

Group 1 was given distilled water (10 ml/kg) intraperitoneally and 1% carboxylmethyl cellulose (CMC) (10 ml/kg) orally and served as the control group. 

Group 2 was given gentamicin (20 mg/kg) and 1% CMC (10 ml/kg) and served as intoxicated and non-treated group. 

Group 3 was given gentamicin (20 mg/kg) and treated with silymarin (100 mg/kg) and served as the reference group. 

Group 4 was given gentamicin (20 mg/kg) and celery extract (25 mg/kg) and served as intoxicated and treated group, low dose. Group 5 was given gentamicin (20 mg/kg) and celery extract (100 mg/kg) and served as intoxicated and treated group, high dose. 

Group 6 was given distilled water (10 ml/kg) and celery (100 mg/kg,) and served as the positive control group. 

**Figure 1:**
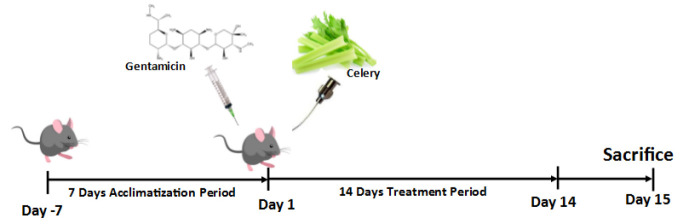
Schematic representation of animal treatment during the *in vivo* study

The timeline showing the treatment protocol is presented in Figure 1.

The body weight of mice was recorded daily. Afterwards, 24 hr after the last treatment, animals were anesthetized by intraperitoneal injection of the mixture of ketamine (100 mg/kg) and diazepam (2 mg/kg) (Fortea et al., 2020) and sacrificed by decapitation. Blood was collected and centrifuged to obtain serum which was used to assess some biochemical indices of liver and kidney functions. The liver, and kidneys were excised, weighed, and used for histological analysis, mRNA extraction, and preparation of 10% organ homogenate.

### Evaluation of biochemical indices of liver and kidneys functions

The effect of celery extract on gentamicin-induced liver and kidneys injuries was assessed through the measurement of serum activities of alanine aminotransferase (ALT_CHRONOLAB_Ref_101-0524) and aspartate aminotransferase (AST_CHRONOLAB_Ref_101-0525), and serum content of creatinine (BIOREX_Ref_BXC0111A) and urea (CHRONOLAB_Ref_101-0274) respectively, using commercial kits.

### Evaluation of oxidative stress markers

Here, 10% (w/v) of liver or kidney tissue homogenate was prepared in ice-cold phosphate buffer and used for the evaluation of oxidative stress markers including lipid peroxidation, GSH content, and superoxide dismutase (SOD) and catalase (CAT) activities as described previously (Misra and Fridovich, 1972; Claiborne, 1985; Djeungoue et al., 2023). 

### Quantitative real-time polymerase chain reaction (qRT-PCR) analysis

The procedure was performed as previously described (Kouam et al., 2020). Relative expression of target genes (*Nrf2* and *HO-1*) was normalized to the endogenous gene (*GAPDH*) used as internal control. The primers sequences of the genes: *Nrf2*, *HO-1*, and *GAPDH* are presented in the supplementary file. 

### Histopathological analysis

Liver and kidneys tissues were fixed in 10% formalin, embedded in paraffin, sectioned into 5-μm thickness and stained with Meyer’s Hematoxylin and Eosin (H&E) for visualization of histological changes under a light microscope.

### Statistical analysis

Data are presented as mean±standard deviation. Comparisons between the mean values of various treatment groups were analyzed by one-way analysis of variance (ANOVA) followed by Bonferroni’s *post hoc* test. Differences between compared groups were considered significant for p<0.05. Analyses were performed using Prism Version 5.03 statistical software (Graph Pad Inc., USA.).

## Results

### Antioxidant activities of celery extracts

Supplementary Figure S1 presents the antioxidant properties of the plant extracts. Overall, both HAG and WAG, as well as vitamin C (VIC), displayed a concentration-dependent effect in scavenging free radicals, reducing power and inhibition of lipid peroxidation. Their EC_50_ values are presented in Table 1. Overall, these results showed that celery extracts possess strong antioxidant properties, comparable to that of VIC. 

**Table 1:** IC_50_/EC_50_ of celery extracts in different chemical antioxidant models

**Extracts**	**Chemical Antioxidant Assay (IC**_50_ **/EC**_50_ ** µg/ml)**					
	DPPH	HO	NO	FRAP	TAC	LP
VIC	3.52±0.8	10.95±1.3	24.35±3.8	4.88±0.9	7.34±1.4	25.27±2.4
HAG	6.65±1.5*	10.75±1.1^ns^	27.59±2.4^ns^	5.23±1.2^ns^	2.99±0.7*	17.56±1.7*
WAG	9.19±1.2*	22.36±2.3*	39.46±2.0*	8.25±1.6*	8.24±1.6^ ns^	18.62±2.2*

(DPPH): DPPH radical scavenging assay; (HO): Hydroxyl radical scavenging assay; (NO): Nitrite oxide radical scavenging assay; (FRAP): Ferric reducing antioxidant power; (TAC): Total antioxidant capacity; (LP): Lipid peroxidation Inhibition assay; Values are means±SD. *significantly different (p˂0.05) compared to VIC. ^ns^significantly non different (p>0.05) when compared to VIC. HAG: Hydro-Ethanolic (30:70, v/v) extract of *A. graveolens*; WAG: Aqueous extract of *A. graveolens*; VIC: Vitamin C

### In vitro hepatoprotective study

#### Gentamicin-induced cell death and disruption of cell membrane integrity in primary mouse hepatocytes

**Figure 2:**
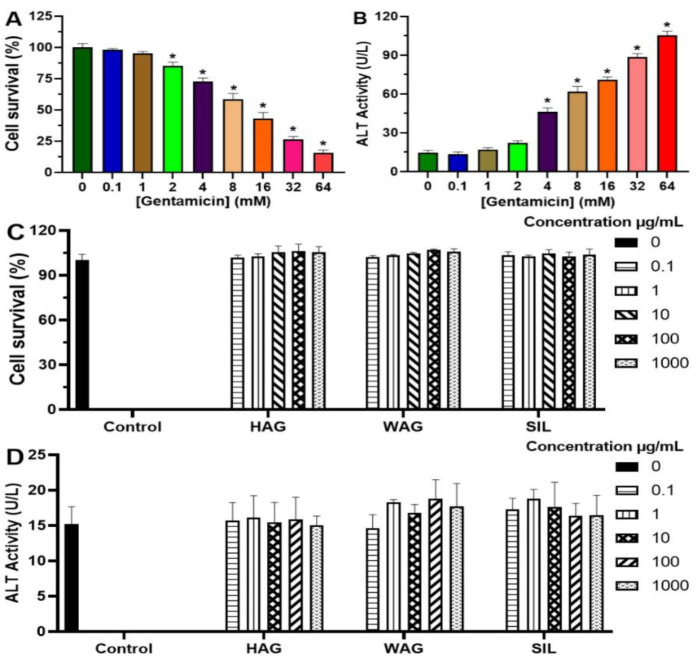
Effect of gentamicin and celery extracts on cell viability and membrane integrity in primary mouse hepatocytes. Primary mouse hepatocytes were treated with different concentration of gentamicin (0.1-64 mM), HAG, WAG or silymarin (0.1-1000 µg/ml) for 12 hr. (A) and (C) Cell viability indicating cytotoxic effect of gentamicin and plant extracts respectively; (B) and (D) ALT activity leakage in the culture medium indicating effect of gentamicin and plant extracts on cell membrane integrity respectively. Values are means ± SD of three independent experiments in triplicate; *p˂0.05, compared to control (0 mM). HAG: Hydro-Ethanolic (30:70, v/v) extract of A. graveolens; WAG: Aqueous extract of A. graveolens; SIL: Silymarin

Gentamicin dose-dependently induced significant (p˂0.05) reduction of cell viability (Figure 2A) and increase in cellular ALT leakage into the incubation medium (Figure 2B). The LC_50_ of gentamicin was 13.45±3.61 mM. Accordingly, 14 mM of gentamicin was considered a toxic concentration to be used for the hepatoprotective studies.

### Celery extracts did not affect cell viability or membrane integrity of primary mouse hepatocytes

The incubation of isolated hepatocytes with HAG, WAG or silymarin (SIL) neither significantly (p>0.05) affected the viability of cells (Figure 1C), nor increased ALT activity in the incubation medium (Figure 1D) at up to 1000 µg/ml, as compared to the untreated cells. 

### Hydro-ethanolic extract of A. graveolens (HAG) attenuates gentamicin-toxicity in primary mouse hepatocytes

**Figure 3:**
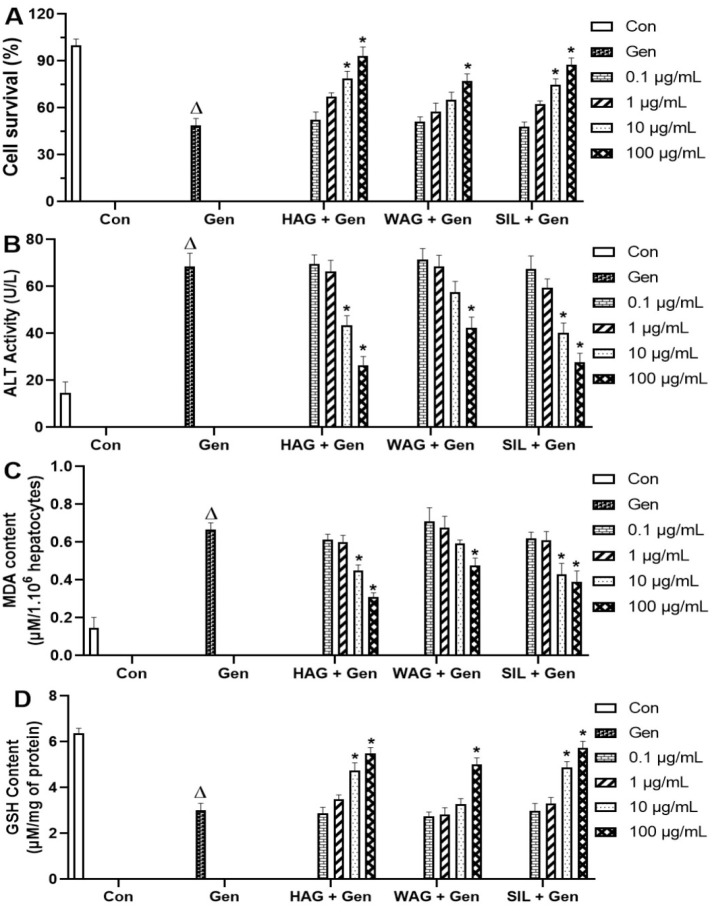
Protective effect of celery extracts against gentamicin-toxicity in primary mouse hepatocytes. Primary mouse hepatocytes were treated with or without gentamicin (14 mM), or simultaneously with gentamicin and HAG, WAG or SIL (0.1-100 µg/ml). After 12 hr of incubation, cell viability (A), ALT activity leakage in the incubation medium (B), MDA (C) and GSH (D) contents in the supernatant of lysed hepatocytes were determined. Values are expressed as means±SD of three independent experiments in triplicate. ^Δ^p˂0.05, values significantly different when compared to the control group (untreated cells). *p˂0.05, values significantly different when compared to the gentamicin group (intoxicated and non-treated cells). HAG: Hydro-Ethanolic (30:70, v/v) extract of A. graveolens; WAG: Aqueous extract of A. graveolens; SIL: Silymarin; Gen: Gentamicin; Con: Control.

The exposure of isolated hepatocytes to gentamicin (14 mM) significantly (p˂0.05) reduced cell viability (Figure 3A), increased ALT activity in the incubation medium (Figure 3B), increased level of MDA content (Figure 3C) and depleted GSH content (Figure 3D) in the cell lysate, when compared to the non-treated cell.

However, co-treatment of cells with HAG, WAG, or SIL (0.1-100 µg/ml) dose-dependently preserved the viability of hepatocytes (Figure 3A), reduced ALT activity in the incubation medium (Figure 3B), attenuated MDA formation (Figure 3C) and restored cellular GSH (Figure 3D). These protective effects were significant (p˂0.05) when HAG or SIL was added at the final concentrations of 10 and 100 µg/ml. Therefore, HAG was considered the more active extract and was tested against gentamicin-induced hepato-renal injuries in mice. 

### In vivo hepato-renal protective study

#### Chronic administration of gentamicin and treatment with HAG did not affect general conditions of mice

During the 14 days of treatment, no death or obvious signs of toxicity (salivation, reduction of locomotion, or dizziness) in different groups of experimental animals was observed. No significant (p>0.05) difference in body weight changes was noted between treated and untreated mice (Supplementary Figure S2A). However, a significant increase in liver weight was noted between gentamicin-intoxicated and treated mice (100 mg/kg) (supplementary Figure S2B). 

### HAG treatment attenuated liver and kidney damage induced by chronic administration of gentamicin in mice

Serum levels of liver function enzymes, ALT and AST, and serum content of kidneys metabolisms, creatinine, and urea, were significantly increased (p˂0.05) in gentamicin-intoxicated and non-treated mice when compared to control (non-intoxicated) mice (Table 2). However, simultaneous treatment of mice with HAG at the dose of 25 or 100 mg/kg or silymarin (100 mg/kg) significantly (p˂0.05) attenuated the increase of serum levels of ALT and AST activities, and creatinine and urea content, when compared gentamicin-intoxicated and non-treated rats. 

**Table 2:** Liver and kidneys function parameters of mice receiving gentamicin and treated with HAG

**Experimental groups**	**Serum biochemical indices of liver and kidneys functions**			
	**Urea(mg/dl)**	**Creatinine(mg/dl)**	**ALT(U/L)**	**AST(U/L)**
Control	27.6±4.7	0.87±0.19	31.7±4.1	49.4±3.9
Gentamicin (20 mg/kg)	54.3±5.1^Δ^	2.91±0.37^Δ^	128.6±5.4^Δ^	145.7±4.6^Δ^
Gentamicin + SIL (100 mg/kg)	42.7±4.4^*^	1.86±0.33^*^	85.4±5.2^*^	98.3±4.9^*^
Gentamicin +HAG (25 mg/kg)	45.1±3.3^*^	2.03±0.25^*^	96.2±6.1^*^	101.3±5.1^*^
Gentamicin + HAG (100 mg/kg)	36.2±4.5^*^	1.12±0.20^*^	63.8±4.5^*^	79.5±4.5^*^
HAG (100 mg/kg)	25.6±3.4^ns^	0.79±0.27^ ns^	34.9±4.6^ ns^	53.7±4.8^ ns^

Data are expressed as means±SD, n=5; ^Δ ^significantly different when compared to the control group (p˂0*.*05); ^ns^non significantly different when compared to the control group (p>0.05); ^*^significantly different when compared to the gentamycin-intoxicated (p˂0.05).

### HAG treatment preserved normal liver architecture in gentamicin-intoxicated mice

**Figure 4:**
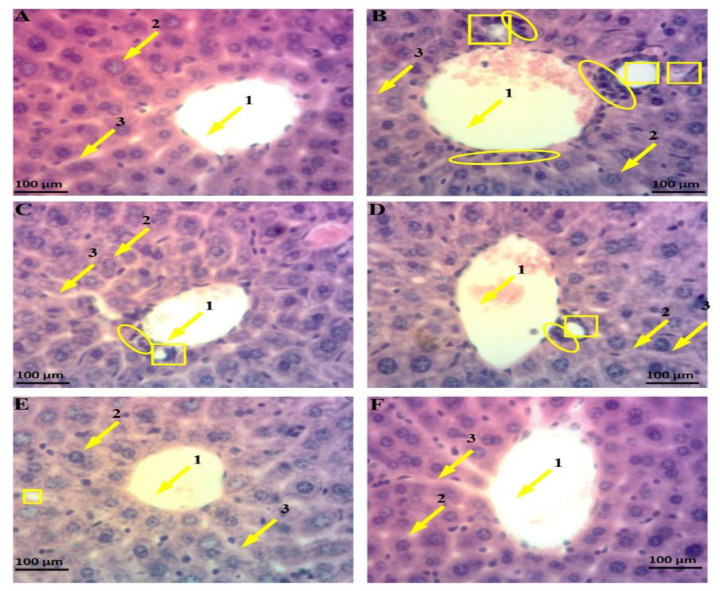
Microphotographs of various experimental groups showing the histopathology of hepatic tissues. Mice were injected intraperitoneally with gentamicin (20 mg/kg bw) and treated by gavage with HAG or silymarin (25 or 100 mg/kg b.w) once per day for 14 consecutive days. 24 hr after the last treatment, mice were sacrificed and the liver was excised, fixed and embedded in paraffin, and sections were stained with hematoxylin-eosin (H-E) and observed under light-microscope, magnificence X-100. (A): Liver slide of control mice (non-intoxicated) showing normal hepatic architecture with centro-lobular vein (arrow 1), hepatocyte (arrow 2) and sinusoid capillary. (B): Liver slide of gentamicin-intoxicated and non-treated mice depicting massive inflammatory cell infiltration (circle) around centro-lobular vein (arrow 1) and severe hepatocytes necrosis (Square). (C) Liver slide of gentamicin-intoxicated mice and treated with silymarin (100 mg/Kg, b.w) presenting nearly normal hepatic architecture with a few hepatocytes necrosis (square) and moderate inflammatory cell infiltration (circle). (D) liver slide of gentamicin-intoxicated mice and treated with HAG (25 mg/kg, b.w) showing mild hepatocytes necrosis (square) and mild inflammatory cell infiltration (circle). (E) liver slide of gentamicin-intoxicated mice and treated with HAG (100 mg/kg, b.w) presenting almost normal hepatic architecture without hepatocytes necrosis and leucocytes infiltration. (F) Liver slide of mice treated with HAG (100 mg/kg, b.w) alone showing normal Liver architecture. HAG: Hydro-Ethanolic (30:70, v/v) extract of A. graveolens

In the control group, microphotograph showed normal hepatic architecture, where the hepatocytes are arranged around the central vein and alternate with blood sinusoids capillary (Figure 4A). Microscopic examination of liver slides from gentamicin-intoxicated mice depicted marked hepatocellular alterations, among which impairment of hepatic lobule architecture, severe hepatocytes necrosis, and massive inflammatory cells infiltration surrounded by the central-lobular vein (Figure 4B). Treatment with HAG (25 mg/kg) or silymarin (100 mg/kg) along with gentamicin exhibited reversal of these changes (Figure 4C and 4D), characterized by a few hepatocytes necrosis and moderate lymphocytes infiltration. However, the protective effects were more obvious at higher doses of HAG (100 mg/kg), as shown by the absence of severe hepatic necrosis or leucocytes infiltration (Figure 4E). 

### HAG treatment maintained normal kidney histology in gentamicin-intoxicated mice

**Figure 5:**
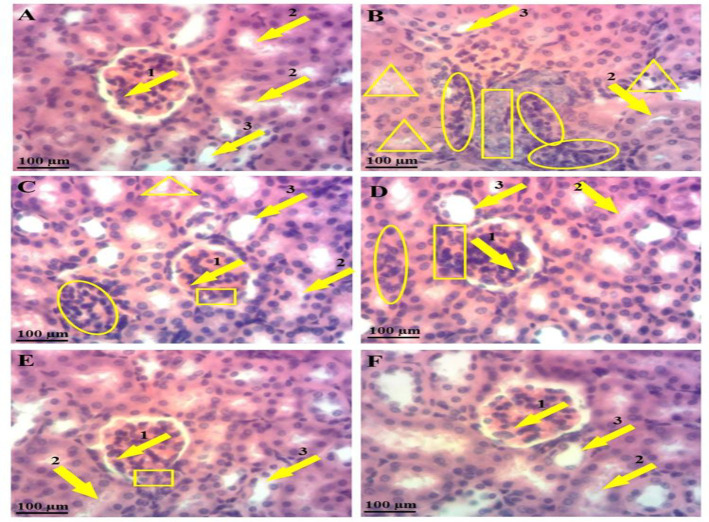
Microphotographs of various experimental groups showing the histopathology of kidney tissues. Mice were injected intraperitoneally with gentamicin (20 mg/kg bw) and treated by gavage with HAG or silymarin (25 or 100 mg/kg b.w) once per day for 14 consecutive days. 24 hr after the last treatment, mice were sacrificed and the kidneys were excised, fixed and embedded in paraffin, and sections were stained with hematoxylin-eosin (H-E) and observed under light-microscope, magnificence X-100. (A): Kidney section of control mice (non-intoxicated) showing normal kidney architecture with glomerulus and Bowman’s capsule (arrow 1), proximal tubules (arrow 2) and distal tubule (arrow 3). (B): Kidney section of gentamicin-intoxicated and non-treated mice depicting massive inflammatory cell infiltration (circle), severe degeneration of glomerulus and Bowman’s capsule (rectangle) and congested proximal tubule (triangle). (C) Kidney section of gentamicin-intoxicated mice and treated with silymarin (100 mg/kg, b.w) presenting nearly normal kidney architecture, with moderated inflammatory cell infiltration (circle) and moderated glomerulus and Bowman’s capsule injuries (rectangle). (D) Kidney section of gentamicin-intoxicated mice and treated with HAG (25 mg/kg, b.w) showing nearly normal kidney architecture, with moderated inflammatory cell infiltration (circle) and moderated glomerulus and Bowman’s capsule injuries (rectangle). (E) Kidney section of gentamicin-intoxicated mice and treated with HAG (100 mg/kg, b.w) showing almost normal kidney architecture, with absence of inflammatory cell and congested proximal tubule, and fewer degeneration of glomerulus and Bowman’s capsule. (F) Kidney section of mice treated with HAG (100 mg/kg, b.w) alone showing normal kidney architecture. HAG: Hydro-Ethanolic (30:70, v/v) extract of A. graveolens

Normal architecture including glomerulus, Bowman’s capsule, and distal and proximal convoluted tubules were observed in kidney tissue of the control mice (Figure 5A). Daily administration of gentamicin markedly disrupted the histology as evidenced by tubular degeneration, severe distal tubular congestion, proximal tubular dilatation, massive inflammatory cell infiltration, and glomerular and Bowman’s capsule injuries (Figure 5B). On the contrary, prevention of these nephron-pathological injuries was observed with simultaneous treatment of gentamicin-intoxicated mice with HAG (25 or 100 mg/kg) or silymarin (100 mg/kg), as evidenced by moderated leucocytes infiltration, absence of tubular congestion and tubular dilatation (Figure 5E), and moderated injury of glomerular and Bowman’s capsule (Figure 5C and 5D).

### HAG treatment attenuates oxidative stress in liver and kidneys tissues of mice receiving gentamicin

**Figure 6:**
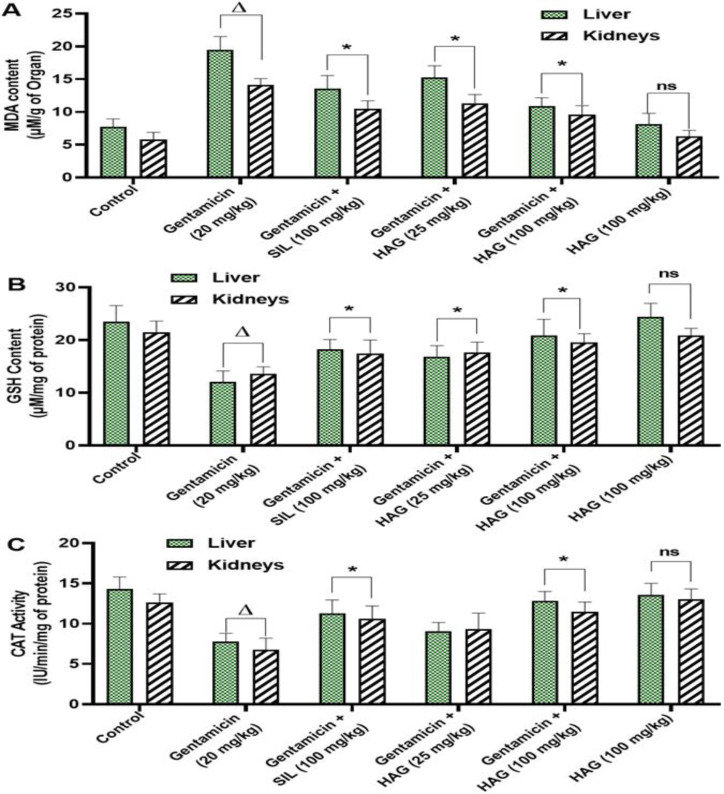
Oxidative stress parameters in liver and kidneys homogenates of mice receiving gentamicin and treated with HAG. Mice were injected intraperitoneally with gentamicin (20 mg/kg b.w) and treated by gavage with HAG or silymarin (25 or 100 mg/kg b.w) once per day for 14 consecutive days. 24 hr after the last treatment, mice were sacrificed and 10% homogenate were prepared from the collected liver and kidneys and used to evaluate the effect of HAG on some oxidative stress parameters: **(A)**: Malondialdehyde (MDA) content; **(B)**: Reduced glutathione (GSH) content; **(C)**: Catalase (CAT) activity; **(D)**: Superoxide Dismutase (SOD) activity. Data are expressed as means ± SD, n = 5; ^Δ ^values significantly different when compared to control group (p˂0.05); ^ns ^values non significantly different when compared to control group (p>0.05); ^*^values significantly different when compared to gentamicin (20 mg/kg) group (p˂0.05) using ANOVA followed by Bonferroni’s post-test. HAG: Hydro-Ethanolic (30:70, v/v) extract of A. graveolens.

MDA content was significantly increased (p˂0.05) in the liver and kidney homogenates of gentamicin-intoxicated and non-treated mice (Figure 6A). Also, the GSH content (Figure 6B), an endogenous antioxidant molecule, as well as the activities of CAT and SOD (Figure 6C and 6D, respectively), were significantly (p˂0.05) reduced in gentamicin-intoxicated and non-treated mice when compared to control mice. However, simultaneous treatment with HAG at the dose of 25 or 100 mg/kg, or silymarin at 100 mg/kg significantly (p˂0.05) restored both hepatic and renal GSH content, and SOD and CAT activities, and decreased MDA content, as compared to gentamicin-intoxicated and non-treated mice.

### HAG treatment increased mRNA expression levels of Nrf2 and HO-1 in the liver and kidney tissues of gentamicin-intoxicated mice

**Figure 7:**
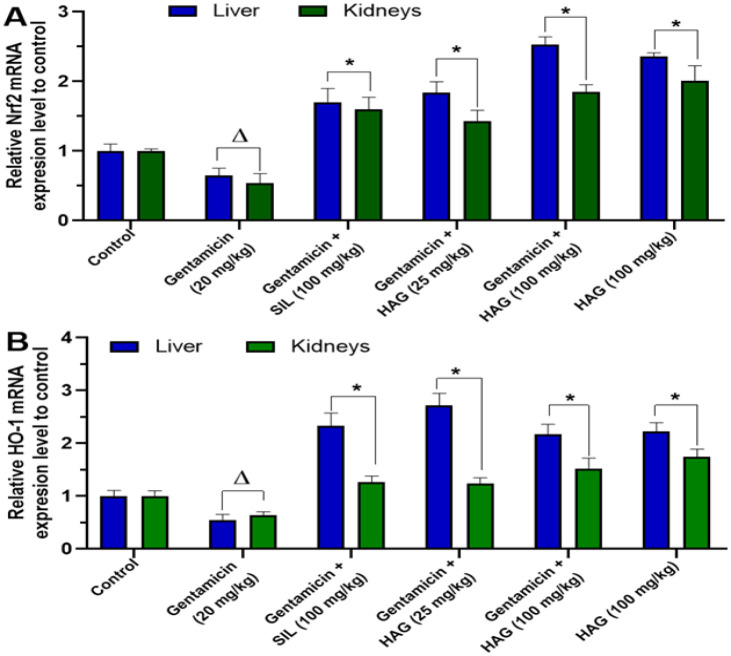
Relative mRNA expression level of Nrf2 and HO-1 in the liver and kidneys tissues of gentamicin-intoxicated mice and treated with HAG. Mice were injected intraperitoneally with gentamicin (20 mg/kg b.w) and treated by gavage with HAG or silymarin (25 or 100 mg/kg b.w) once per day for 14 consecutive days. 24 hr after the last treatment, mice were sacrificed and total RNA was extracted from the liver and kidneys; then relative mRNA expression level of Nrf2 **(A)** and HO-1 **(B)** were determined by qRT-PCR. GAPDH was used as internal control. Data are expressed as means ± SD, n = 5; ^Δ ^values significantly different when compared to control group (p˂0.05); ^ns ^values non significantly different when compared to control group (p>0.05); ^*^values significantly different when compared to gentamicin (20 mg/kg) group (p˂0.05) using ANOVA followed by Bonferroni’s post-test. HAG: Hydro-Ethanolic (30:70, v/v) extract of A. graveolens.

As presented in Figure 7, chronic administration of gentamicin alone for 14 consecutive days significantly reduced the mRNA levels of *Nfr2* (Figure 7A) and *HO-1* (Figure 7B) in the liver and kidneys. In HAG co-treated animals, at the dose of 100 mg/kg, the mRNA level of *Nrf2* (Figure 7A) was increased by up to 2.5-fold as compared to the untreated mice. Similarly, the mRNA level of *HO-1* (Figure 7B) was also increased by up to 2.2-fold.

## Discussion

Hepatotoxicity and nephrotoxicity are two major drawback effects that reduce the efficiency of gentamicin, especially when sustained administration is required (Chaves and Tadi, 2022). It is therefore necessary to search for drugs to abolish their toxicity. Hence, we tested the ability of extracts of celery to prevent hepato-renal toxicity induced by gentamicin. The implication of oxidative stress in the pathogenesis of gentamicin-induced hepato-renal injury is well established (Khan et al., 2011). Accordingly, substances that can inhibit oxidative stress-mediated injury could be of great importance for the prevention of these side effects. In this study, two extracts, aqueous (WAG) and hydro-ethanolic (HAG) were prepared from *A. graveolens*, and their antioxidant activities were evaluated *in vitro*. Oxidative stress being a complex physiological process, several experimental models are necessary to study the antioxidant properties of plant extracts (Blažeković et al., 2010; Fu et al., 2011). Hence, this activity was tested in free-cell systems through measurement of the capacity of our plant extract to scavenge DPPH°, hydroxyl (HO°), and nitric oxide (NO°) radicals, reduce ferric ion (Fe^3+^) and phosphomolybdenum complex, and inhibit lipid peroxidation. 

Overproduction of free radicals is associated with the pathogenesis of various xenobiotics toxicity (Hagerman et al., 1998; Jaeschke et al., 2003; Pandey and Rizvi, 2009). Therefore, scavenging DPPH, HO°, and NO° radicals appears as one of the best barometers to assess the antioxidant potential of plant extracts. In our study, both HAG and WAG, as well as vitamin C (VIC), effectively scavenged DPPH, HO°, and NO° free radicals, suggesting that celery extracts can give an electron or hydrogen atom to scavenge free radicals. The reducing power of plant extracts can also serve as a substantial indicator of the antioxidant potential (Rao et al., 2010). Accordingly, we evaluated the ability of HAG and WAG to reduce Fe^3+^ and phosphomolybdenum to Fe^2+^ and phosphomolyb-2-molybdic complex, respectively. HAG extract displayed the highest reducing ability, as shown by its respective EC_50_, 5.23, and 2.99 µg/ml, which were comparable to that of standard VIC, 4.88 and 7.34 µg/ml, respectively (Table 1). Lipid membrane peroxidation is also an important pathological process involved in xenobiotics-induced tissue damage (Costa et al., 2011). In this study, both extracts and VIC were found to inhibit lipid peroxidation *in vitro*. HAG displayed the highest inhibitory activity, with an IC_50_ of 17.56 µg/ml (Table 1). From these observations, it can be suggested that celery extracts can scavenge HO° radical and/or chelate iron to prevent lipid peroxidation.

The negative effect of gentamicin on the liver and kidneys are well recognized. Experimentally, extracellular level of transaminases activity is routinely used to evaluate hepatotoxicity, while serum content of creatinine and urea is widely considered a criterion to assess kidneys functions (Khan et al., 2011; Chaves and Tadi, 2022). Prior to assessing the hepato-renal protective effect of celery extracts in animal models, their ability to protect primary mouse hepatocytes from gentamicin-induced oxidative damage was tested. A concentration-response study was conducted to determine the LC_50_ of gentamicin. Our result showed that at 14 mM, gentamicin induced hepatotoxicity in primary mouse hepatocytes, characterized by the decrease of cell survival to nearly 50% and the increased activity of ALT in the incubation medium. This viewing could be attributed to the toxic effect of gentamicin, which was further endorsed by the significant (p˂0.05) increase of MDA content, an indicator of lipid membrane peroxidation, and the depletion of cellular GSH content, indicator of oxidative stress development. In contrast, simultaneous treatment of hepatocytes with HAG or silymarin serving standard hepatoprotective compounds, at 10 or 100 µg/ml respectively, significantly (p˂0.05) maintained cell survival, reduced the extracellular activity of ALT, inhibited the excessive formation of MDA, and restored GSH content. This finding suggested that HAG could interfere with the mechanisms by which gentamicin induces cell death, and consequently, protect hepatocytes from gentamicin-induced hepatotoxicity. However, in WAG co-treated hepatocytes, relative protection was observed only at 100 µg/ml, suggesting that HAG was the more active extract, and was therefore selected for the in *vivo* study. 

In this animal model of gentamicin-induced hepato-renal injury, we observed that administration of gentamicin for 14 consecutive days yielded an abnormal increase in serum level of transaminases, creatinine, and urea content, suggesting hepatic and renal injuries induced by gentamicin. These observations were further supported by histopathological changes observed in the liver and kidneys tissues of gentamicin-intoxicated and non-treated mice, evidenced by severe hepatocytes necrosis, massive infiltration of leucocytes, disruption of hepatic lobule architecture, or tubular congestion and glomerular injury. However, simultaneous treatment with HAG or silymarin, at the doses of 25 or 100 mg/kg, effectively inhibited gentamicin-induced elevated serum levels of hepatic and renal function biomarkers. In line with these findings, the histopathological examination also showed an attenuation or full abrogation of liver and kidney damage by concomitant administration of HAG. Our result, therefore, suggested that HAG protects gentamicin from inducing hepatic necrosis and kidney dysfunctions in mice. We also found that HAG (100 mg/kg) alone has no significant effect on the variation of body weight, as compared to the control mice. Also, no evidence of liver or kidney injuries was noted, as confirmed by the level of hepatic and renal function biomarkers, and the histological analysis. From these results, it can be inferred that HAG at 100 mg/kg, is non-toxic for the mice. 

Although not fully elucidated, oxidative stress-mediated damage is highly involved in the mechanism of gentamicin toxicity (Cuzzocrea et al., 2002; Khan et al., 2011). Usually, cells possess antioxidant defense systems, composed of antioxidant enzymes like CAT and SOD, and antioxidant molecules such as GSH, for protecting themselves against oxidative stress. This antioxidant defense system is regulated by nuclear factor erythroid 2-related factor-2 (*Nrf2*), a transcription factor that regulates the expression of antioxidant enzymes, including Hem oxygenase-1 (*HO-1*), CAT, and SOD by binding to the antioxidant response element (ARE) in their promoter region (Shin et al., 2013). Here, qRT-PCR revealed that administration of gentamicin in mice significantly (p˂0.05) reduced the mRNA expression level *Nrf2* and its target gene *HO-1* in the liver and kidneys, as compared to non-intoxicated mice. A significant (p˂0.05) decrease of SOD and CAT activities, and GSH content in the liver and kidneys homogenates of gentamicin-intoxicated mice was also observed. In contrast, co-treatment with HAG or silymarin increased the liver and kidneys *Nrf2 *mRNA expression level by up to 2.5-fold, concomitantly with a significant (p˂0.05) increase of *HO-1* mRNA expression level. Moreover, the significant (p˂0.05) decrease of CAT and SOD activities, and GSH content were fully overturned when mice were co-treated with HAG (Figure 6). These findings suggest that HAG could protect mice from gentamicin-induced hepato-renal toxicity, by restoring endogenous antioxidant capacities, through up-regulation of the Nrf2-signaling pathway and its target antioxidant genes. 

This study has potential limitations. For example, the sample size in the animal study was relatively small, with only thirty mice divided into six groups. Also, the possible protective mechanism of HAG has been investigated at the transcriptional level only using qRT-PCR. Accordingly, it would be more interesting if the protective mechanisms on the target genes could be assessed at the post-translational level through western blot analysis. Moreover, investigating the nuclear translocation of *Nrf2* transcription factor could offer a better approach to understand the molecular mechanism underlying the protective action of HAG.

In summary, the present study reports the ability *A. graveolens* extract to protect liver and kidney damages induced by 14-days administration of gentamicin through antioxidant mechanisms. The aqueous extract (WAG) and hydro-ethanolic (30:70, v/v) extract (HAG) of *A. graveolens* were tested. For the *in vitro* study, our results demonstrated that both extracts exert strong chemical antioxidant activities and hepatoprotective effect against gentamicin-induced oxidative damage in primary mouse hepatocytes, the most active being HAG. *In vivo*, HAG displayed protective effect against gentamicin-induced hepato-renal toxicity in mice. The activation of the *Nrf2*-antioxidant signaling pathways, through up-regulation of the mRNA expression levels of *Nrf2* and *HO-1*, increasing of endogenous antioxidant capacities, and attenuation of oxidative stress damage can be considered the major mechanisms by which HAG alleviates gentamicin-induced liver and kidney toxicity. However, further investigations are warranted to explore the bioactive chemicals in HAG to develop therapeutics for the treatment of xenobiotics-induced hepato-renal dysfunctions. 

## Abbreviations

ALT: Alanine aminotransferase

AST: Aspartate aminotransferase

CAT: Catalase

CMC: Carboxyl-methyl cellulose

DPPH: 2,2-Diphenyl-Picryl-Hydrazyl

FRAP: Ferric reducing antioxidant power

GEN: Gentamicin

GSH: Reduced glutathione

HAG: Hydro-ethanolic (30:70, v/v) extract of *Apium graveolens*

*HO-1*: Heme-oxygenase-1

LPO: Lipid peroxidation

MDA: Malondialdehyde

MTT: 3,4,5-Dimethylthiazol-2-yl)-2,5-Diphenyltetrazolium Bromide

*Nrf2*: Nuclear factor erythroid 2-related factor-2

SIL: Silymarin

SOD: Superoxide dismutase

TAC: Total antioxidant capacity

VIC: Vitamin C

WAG: Aqueous extract of *Apium graveolens*
